# Image post-processing for SILMAS: structured illumination light sheet microscopy with axial sweeping

**DOI:** 10.1364/BOE.531210

**Published:** 2024-07-30

**Authors:** David Frantz, Courtney J. Wright, Allison J. Schaser, Deniz Kirik, Elias Kristensson, Edouard Berrocal

**Affiliations:** 1 Division of Combustion Physics, Department of Physics, Lund University, Lund, Sweden; 2 Brain Repair and Imaging in Neural Systems (B.R.A.I.N.S) Unit, Department of Experimental Medical Science, Lund University, BMC D11, 22184 Lund, Sweden; 3 Department of Speech, Language, & Hearing Sciences, Purdue Institute for Integrative Neuroscience 207 S. Martin Jischke Dr., DLR 335, Purdue University, West Lafayette, IN 47907, USA

## Abstract

In this article, we propose a post-processing scheme for the novel volumetric microscopy technique SILMAS. We demonstrate this scheme on data from an alpha-synuclein transgenic mouse brain. By combining structured illumination and axial sweeping, a SILMAS measurement provides a prerequisite for quantitative data extraction through improved contrast and optical sectioning. However, due to the technique’s efficient removal ofb multiple scattered light, image artifacts such as illumination inhomogeneity, shadowing stripes, and signal attenuation, are highlighted in the recorded volumes. To suppress these artifacts, we rely on the strengths of the imaging method. The SILMAS data, together with the Beer-Lambert law, allow for an approximation of real light extinction, which can be used to compensate for light attenuation in a near-quantitative way. Shadowing stripes can be suppressed efficiently using a computational strategy thanks to the large numerical aperture of an axially swept light sheet. Here, we build upon prior research that employed wavelet-Fourier filtering by incorporating an extra bandpass step. This allows us to filter high-contrast light sheet microscopy data without introducing new artifacts and with minimal distortion of the data. The combined technique is suitable for imaging cleared tissue samples of up to a centimeter scale with an isotropic resolution of a few microns. The combination of a thin and uniform light sheet, scattered light suppression, light attenuation compensation, and shadowing suppression produces volumetric data that is seamless and highly uniform.

## 1. Introduction

Detailed visualization of three-dimensional (3D) connectivity in the mouse brain is an important goal in neurobiology. This concerns the study of neurodegenerative diseases, such as the prion-like propagation of alpha-synuclein (aSyn) aggregates in Parkinson’s disease [1,2] and the accumulation of amyloid beta plaques and neurofibrillary tangles in Alzheimer’s disease [3,4]. Volumetric imaging of brain tissue from mouse models of neurodegenerative diseases provides valuable information on disease progression and may indicate potential targets for therapeutic interventions.

The use of fluorescent labels fused to the proteins of interest gives targeted contrast that helps to visualize the distribution of pathology. However, the highly scattering nature of brain tissues still makes high-contrast imaging of such samples highly challenging. In the last decade, volumetric imaging with micron resolution of fluorescent tissue samples have come to rely on the use of tissue clearing combined with light sheet fluorescence microscopy (LSFM). Tissue clearing techniques [5–7] homogenizes and render biological samples transparent by chemically removing the lipid content. Thereby, visible light is able to penetrate centimeters of biological tissue which allows for imaging of the entire mouse brain. These opportunities demand LSFM modalities which enable equally large field of view (FOV) with thin and uniform optical sectioning.

Despite tissue clearing, traditional LSFM techniques often suffer from reduced optical sectioning and contrast deeper into large samples as a result of sample-induced light scattering. Several LSFM techniques that optically suppress light scattering have been proposed, including confocal ultramicroscopy [8], structured illumination (SI-LSM) [9], confocal slit detection [10], and confocal light sheet microscopy [11]. These techniques suppress scattered light efficiently while requiring modest computational power compared to methods that rely on data deconvolution [12,13]. In general, however, they mainly operate in a single direction. In our recent article [14] we introduced structured illumination light sheet microscopy with axial sweeping (SILMAS), which combines the strengths of structured laser illumination planar imaging (SLIPI) [15,16] and axial sweeping light sheet microscopy (ASLM) [17]. This new technique can achieve more complete suppression of light scattering while also providing microscopy on a very large FOV.

**Fig. 1.**
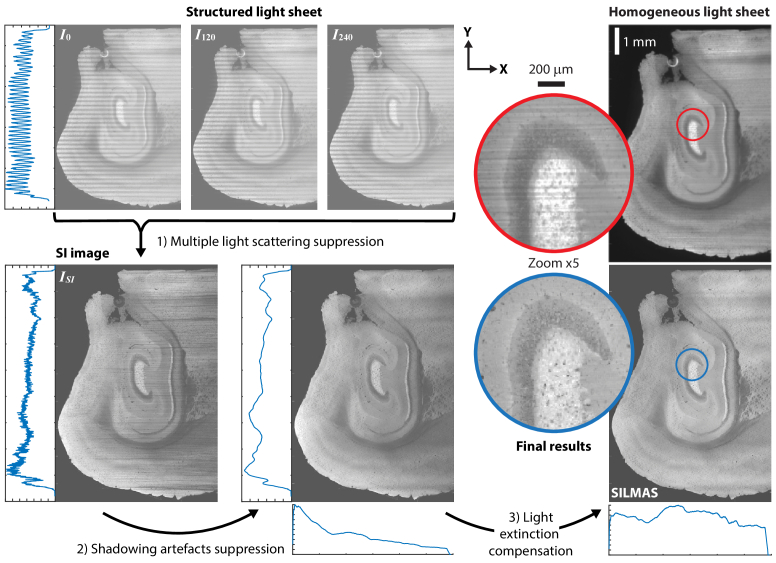
Illustration (in 2D) of the pipeline for volumetric SILMAS image post-processing.

After the suppression of light scattering, data recorded with these techniques require post-processing in order to fulfill their potential for detailed data analysis and 3D visualization. In this work, we propose a multi-step image post-processing scheme, illustrated in Fig. 1, which addresses image artifacts in SILMAS. Although the work is focused on SILMAS, individual steps of the post-processing scheme are valid for similar imaging modalities.

One persistent issue in light sheet microscopy is attenuation of the signal deeper into the tissue, reducing visibility and signal-to-noise ratio (SNR). The attenuation is mainly due to absorption and scattering of the excitation light as it propagates in the tissue. This issue is more visible after optical suppression of scattered light, as induced blur otherwise conceals the underlying problem.

In tissue with near-homogeneous optical properties, the attenuation of excitation light along the propagation axis will follow an exponential decay in accordance with the Beer-Lambert law [18–20]. For imaging modalities with complete rejection of scattered light, the fluorescence signal will exclusively originate from this plane. In our post-processing scheme, we utilize this to compensate for attenuation in a near-quantitative way. Combined with a large FOV and uniform optical sectioning, this scheme can provide comparable intensities throughout the entire sample.

Another challenge for light sheet imaging techniques is shadowing artifacts [21]. They originate from the scattering and/or absorption of relatively large objects in the sample that significantly block the propagation of light, creating dark shadow lines behind them. This is especially prominent for thin light sheets. Multiple strategies to suppress shadowing have been suggested, and, broadly, they can be separated into optical [22–24] and computational solutions [25–31].

The optical strategies aim to increase the angular variation of the incoming light so that no region is completely shadowed. Simple implementations include multi-angle illumination, where the sample is illuminated from two or more directions, either sequentially or simultaneously [22]. More advanced implementations include light sheet pivoting, where the incident angle of the light is rapidly swept over a narrow angular band in the illumination plane [23]. Such techniques are difficult to combine with structured illumination, where precise control over the illumination profile is needed [24]. ASLM techniques natively possess some optical shadowing suppression due to the high numerical aperture (NA) light sheet which introduces a broader range of incident angles transverse to the illumination plane. Although highly potent, optical shadowing suppression typically increases complexity of the optical setup, with possible misalignment issues and acquisition time as a result.

Computational strategies instead rely on image post-processing to compensate for shadowing artifacts. Methods include modeling light propagation [25], variational stationary noise remover algorithms (VSNR) [26,27], Fourier filtering [28,29], and refined or hybrid Fourier filtering methods [30,31]. These methods aim to visually even out the intensity profile of an image, but generally do not compensate for the loss of real contrast and SNR. Therefore, they may be insufficient in cases of strong shadowing where little to no signal exists within the shadow. However, in combination with an optical strategy to partially suppress shadowing, a computational approach can yield much improved datasets. For SILMAS data, the native shadowing suppression from axial sweeping makes computational suppression an attractive complement. In addition, post-image suppression of shadowing artifacts helps suppress structured noise from structured illumination.

Our method for suppressing shadow artifacts is based on wavelet-Fourier filtering, originally suggested by Münch et al. [30] to compensate for dead pixels in electron microscopy and tomography. In their paper, Münch et al. showed that their filtering scheme could remove striping artifacts while minimally affecting the image. This scheme, with some modifications, has since been successfully used for suppressing shadowing structures in tissue by Zhang et al. [32]. We again modified the scheme to accommodate high contrast imaging over a FOV covering varying regions.

In this paper, we describe large FOV volumetric imaging and post-processing which provide artifact-free data with comparable intensities across the entire volume. This is achieved by acquisition and image processing of SILMAS data, including sample preparation, imaging, calibration, shadowing suppression, and extinction calibration. Ultimately, the refined data will provide the best foundation for quantitative analysis of the spatial distribution, size, and characteristics of pathological features in brain tissue, which are difficult to achieve in such detail with other imaging methods.

## 2. SILMAS imaging of mouse brain tissue

### 2.1. Theory of SILMAS

Light sheet microscopy with SILMAS, detailed in [14], enables volumetric imaging with high-contrast, thin and uniform optical sectioning, with centimeter-scale FOV. To achieve this, the light sheet intensity is modulated with a sinusoidal structure transverse to the propagation axis. For each plane, three images, where the modulation is phase-shifted 120 degrees between each, are recorded using axial sweeping. Here, the focused region of the light sheet is swept across the FOV, creating an elongated focus. Using the differences between the images, it is possible to suppress their unmodulated component, which consists of unwanted multiply scattered and out-of-focus light, while keeping the wanted signal [18–20].

The three structured images, $I_{0}$ , $I_{120}$ and $I_{240}$ , with the modulation along the Y axis (see Fig. 1), are recorded for each XY plane and combined into the image $I_{SI}$ using:

(1) $$I_{SI}=\frac{\sqrt{2}}{3}\sqrt{{\left(I_{0}-I_{120}\right)}^{2}+{\left(I_{0}-I_{240}\right)}^{2}+{\left(I_{120}-I_{240}\right)}^{2}}$$

For the case of a homogeneous illumination, where the modulation of the light intensity is discarded, the conventional light sheet image $I_{C}$ can be mathematically deduced such as:

(2) $$I_{C}=\frac{I_{0}+I_{120}+I_{240}}{3}$$

### 2.2. Description of the optical setup

**Fig. 2.**
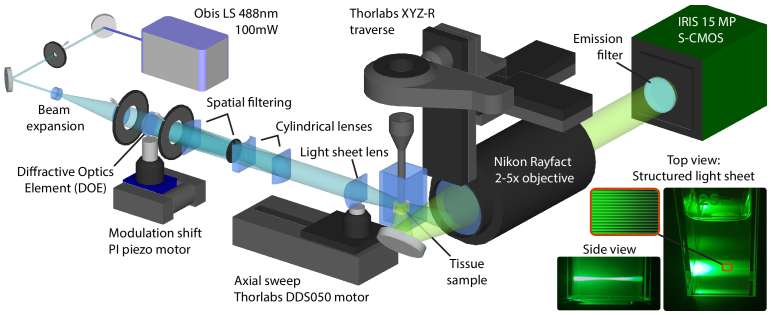
Optical setup for SILMAS imaging. The horizontally oriented structured light sheet is imaged from below as the sheet waist is swept along the propagation direction.

The optical setup is shown in Fig. 2. It has a 488 nm 100 mW diode laser (OBIS LS from Coherent). The beam is expanded to 25 mm before a sinusoidal modulation is imposed in the transverse direction, creating lines in the propagation direction. The modulation is formed by letting a custom diffractive optical element (from Holo/Or) separate most of the beam’s intensity into two first-order diffraction components. A 50 mm focal cylindrical lens focuses the beam onto a 3D-printed spatial filter which blocks any other diffraction orders. The two diffraction orders are focused to infinity in each axis by a pair of cylindrical lenses creating a 15 lp/mm sinusoidal line structure that is preserved over the full FOV. The modulated beam is focused into a horizontal light sheet by a cylindrical lens with NA 0.1. The modulation is phase-shifted by a piezo controller (from Physik Instrumente) that displaces the diffractive element.

The axial sweeping is performed by physically displacing the light sheet waist in the propagation axis (X-axis) by means of a motorized cylindrical lens (DDS050 servo motor from Thorlabs). The movement is synchronized with the camera shutter readout window, ensuring that only the signal from the focused region of the light sheet is collected. The width of the window specifies the length of that region and, in turn, the Z-axis width of the probed volume in the sample.

The efficiency of axial sweeping is highly dependent on the accuracy of the synchronization between the sheet waist and the read-out window. Thus, the calibration of the sweep speed is critical. The sweep speed of the camera read out-window (in pixels/s) is a linear function of the exposure time, the window width, and the readout time and is set based on desired signal strength and Z-axis resolution. To match the light sheet movement with the desired speed, the pixel position of the sheet waist in the image is measured as it is displaced in the sample. From the deduced positions, an exact time-position relation for the sweep motor during a sweep is established. The calibration compensates for any imprecise refractive index or magnification. To synchronize the acquisition with the motor movement, the camera is triggered on motor movement by the motor controller.

The sample is attached, using a tissue adhesive, to the bottom of a 3D printed holder that is connected to the scanning stage through a magnetic snap-in mechanism. In addition to vertical Z-stacking, the scanning stage has X, Y and rotational stepper motors for positioning and tiling (from Thorlabs). The sample and holder are submerged in an RI-matching mounting solution in a 25×25×50mm glass cuvette (from Hellma). The fluorescent signal passing through the bottom of the cuvette is directed by a mirror and is collected by a 2-5X objective with NA 0.15 (Rayfact from Nikon) and an Iris 15 sCMOS camera (from Teledyne Photometrics) with a 520/40 nm emission filter (from Semrock). The absorption and fluorescence emission peaks of eGFP are approximately 488 and 510 nm, respectively. Rather than a traditional microscope objective, the imaging lens for this setup is chosen for its low distortion and sensitivity over a large FOV, ensuring comparable signal from the entire sample.

### 2.3. Preparation of the sample

All mice were cared for by the Purdue University Laboratory Animal Program in an Association for Assessment and Accreditation of Laboratory Animal Care (AALAC) accredited vivarium. The procedures included in this study were performed in accordance with a protocol approved by the Purdue University Animal Care and Use Committee (IACUC 2008002069, approved October 2020).

The tissue samples prepared in this manuscript are from individual brain hemispheres from aged (10 - 25 months old) aSyn transgenic mice. This mouse model endogenously expresses human aSyn, fused to eGFP (SynGFP), with an aggressive disease-associated point mutation that causes a substitution with threonine amino acids (Alanine 53 GCA > ACA) leading to abnormal protein aggregation. Endogenous SynGFP is present in neuropil as puncta and with a homogeneous signal in a large subset of cell bodies. These mice were injected with either monomeric aSyn to act as a control, or preformed fibrils of untagged aSyn (aSyn PFF), which induces aggregation of aSyn. Therefore, both endogenous and aggregated SynGFP are present within the specimen. Aggregated aSyn plays an integral role in the development of age-related neurodegenerative disorders, such as Parkinson’s disease, where it can be found in the form of Lewy bodies [33]. The SynGFP signal in this model has been previously characterized both at baseline and following injection of aSyn PFF [34].

The untagged aSyn PFF injection was prepared according to previously published protocols [35,36]. Fibrils were prepared in reactions (200 *μ*l per tube) containing 360 *μ*M (5 mg/ml) aSyn monomer in assembly buffer (50mM Tris/100mM NaCl, pH 7.0). They were incubated at 37^∘^C with constant agitation (1000 rpm) in an orbital mixer. The reactions were stopped after 5 days, then aliquoted and stored at 80^∘^C until use. Immediately prior to injection, the PFF preparations were briefly sonicated. The animal was anesthetized (isoflurane 1-2%) and injected with 1.5 *μ*l (5 mg/ml) of freshly sonicated PFFs or monomeric aSyn using a stereotactic injection into the right striatum (coordinates from Bregma; AP,-0.5 mm; ML, -2.0 mm, and dural surface; DV, -2.6 mm). Following injection, the animal was returned to its home cage.

Mice were euthanized by overdose of anesthesia (isoflurane) followed by rapid decapitation at 2 or 4 weeks following injection with aSyn. The mouse brain was excised immediately post-mortem and placed into a vial of 6 ml of fresh 4% PFA in PBS. The brain was fixed using a Pelco Biowave Pro for 90 min at 150 W in a 30^∘^C circulating water bath. Then it was moved to 4 ^∘^C to continue fixation overnight. The PFA was replaced the next day with 0.05% sodium azide in PBS, in which the brain was stored at 4 ^∘^C pending the clearing procedure.

Just prior to the clearing, the brain was hemisected and washed in fresh 0.1M PBS for 5 minutes. The hemispheres were then fully immersed in X-CLARITY^™^ hydrogel solution, containing a mixture of Polymerisation Initiator in 0.1M PBS, and a Hydrogel Solution mixture as per the kit instructions, and left to incubate at 4^∘^C for 24 hours. Subsequently, the hemispheres underwent polymerisation in the same hydrogel solution at -90 kPa for 3 hours at 37^∘^C, and were then rinsed with X-CLARITY^™^ electrophoretic tissue clearing (ETC) solution. Samples were next subjected to active lipid removal via electrophoresis for 56 hours, at 0.5 A, 37^∘^C, and a pump speed of 50 rpm, using the X-CLARITY^™^ Tissue Clearing System.

Finally, the cleared tissues underwent three 24-hour washes in PBS-T (Triton 0.2%) at ^∘^C to ensure complete removal of any residual sodium dodecyl sulfate (SDS) solution. Prior to imaging, the brains underwent three 5-minute washes with distilled water to remove residual PBS-T and were placed into a refractive index matching solution (RIMS) made from 88% HistoDenz [37] overnight at room temperature. This solution was changed with fresh RIMS immediately before imaging.

### 2.4. Data collection

**Fig. 3.**
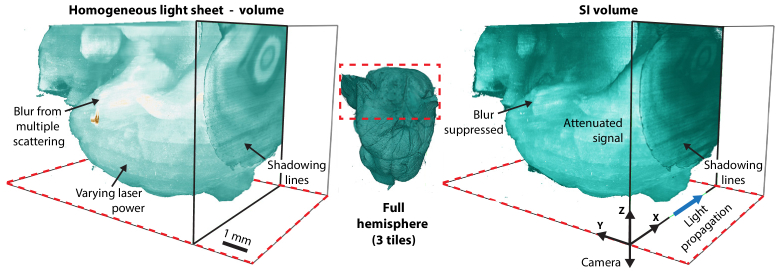
Unfiltered data volumes with and without utilising the structured light sheet. Both volumes have prominent shadowing in the propagation direction. The structured data in particular show strong light attenuation while the non-structured data suffers from scattered signal, including strong reflections from an air bubble trapped within the sample. The layers of intensity in the Z-direction are from incremental increase in illumination power.

For the imaging objective, the magnification was set at 2.125 and the NA to 0.15. The size of the sweep window defined a light sheet thickness of 4 *μ*m, with NA 0.1. Scans were carried out in the Z-direction at three positions over approximately 3000 planes with a step size of 2 *μ*m. Two Z-stacks were created per position; a homogeneous light sheet stack and a structured light sheet stack, of approximate dimensions 6×8×6mm with isotropic 2 *μ*m^3^ voxels. With the camera’s 16-bit image depth this corresponds to about 82 GB/stack. The two stacks from one position are shown in Fig. 3 together with an overview of the full hemisphere from merging the three structured stacks.

The data in Fig. 3 shows a number of imaging artifacts which reduces the overall quality. The homogeneous data suffers from resolution degrading blur from multiple scattering, visible as a bright glow. When the blur is suppressed by utilising the structured illumination imaging, extensive signal attenuation is revealed along both the X- and Z-axis. To limit this, illumination power was incrementally increased linearly with Z-position during acquisition, which is visible as layers in the data. The data in both stacks also show shadowing artifacts from objects that significantly obtrudes the light path. Additionally, imaging techniques that use structured light sheets may have residual line structures from the modulation in cases where beam steering or intensity mismatch have introduced differences between composite images.

### 2.5. Overview of the data post-processing

The focus of the post-processing scheme is to improve the SI volume data by targeting the identified data artifacts. A subset of the unfiltered data volume is available in Dataset 1, Ref. [38]. The implementation of the post-processing scheme is done in MATLAB and can be found in Code 1, Ref. [39] Code 2, Ref. [40] and Code 3, Ref. [41] .The two main processes within the scheme are shadowing suppression (Code 3 [41]) and light extinction compensation (Code 1, Ref. [39]). Additionally, as a prerequisite step for both processes, the tissue is segmented in the image (Code 2, Ref. [40]). While utilized in the shadowing suppression algorithm, the segmentation plays a more integral role in the light extinction compensation algorithm, and the two algorithms also share the same theoretical background. Therefore, these processes are discussed jointly.

**Fig. 4.**
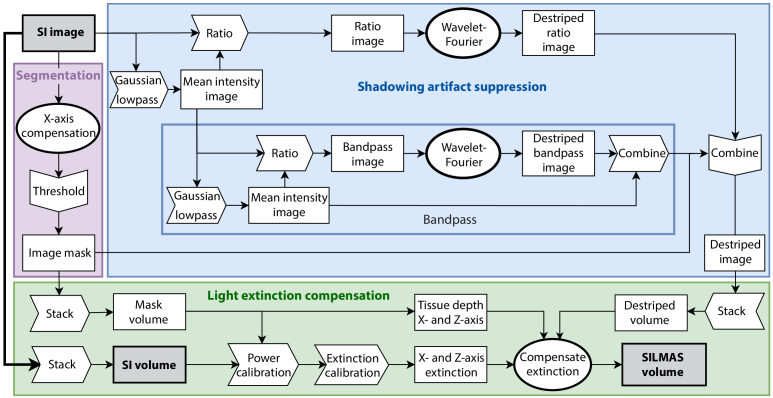
Flowchart of the full post-processing scheme. The segmentation (purple) and shadowing suppressing (blue) are performed in 2D, while extinction compensation (green) is done in 3D. The bandpass process is an optional expansion on our shadowing suppression method.

The segmentation and shadowing suppression are both performed in 2D on an image-by-image basis, while the extinction compensation requires the full data stack. However, the underlying calibration calculations for the extinction compensation are performed on the data before shadowing suppression. Thus, segmentation is performed as step one, shadowing suppression and extinction calibration can be performed in parallel, but the extinction compensation is applied at the end. Figure 4 shows the flowchart of the full post-processing scheme, demonstrating the interconnections and overarching logic between the color-coded main processes and their various sub-processes.

The size of volumetric datasets generated from a single sample can range into the Terabyte range. To accommodate large sets, the code is written to only hold a fraction of the data in memory at any given time, which means reading the data from the hard drive several times. For this reason, the code is much more efficient when working with data stored on an SSD drive. We ran the post-processing code on a PC with an Intel Core i7-7800X CPU at 3.50 GHz, 64 GB of RAM and NVIDIA GeForce GTX 1080 GPU with 8 + 32 GB (dedicated + shared) GPU memory. The full algorithm was processing data at 15 GB / hour, with the shadowing suppression alone running at 35 GB / hour, the extinction compensation at 47 GB / hour and the segmentation at 63 GB / hour.

## 3. Shadowing artifacts suppression

### 3.1. Description of the method

Our filtering scheme for the suppression of shadow artifacts in Code 3, Ref. [41] is performed in 2D on an image-by-image basis and builds upon wavelet-Fourier filtering. It is implemented in a MATLAB environment based on the original code provided by Münch et al. [30]. Our scheme is especially suitable for high-contrast LSFM modalities with varying intensity regions within the FOV. For SILMAS in particular, there is additional synergy in using computational shadowing suppression, such as optical suppression from ASLM and potential line artifacts from the illumination structure. However, shadow artifacts are common in many types of light sheet imaging, and our implementation is stand-alone and can in principle be used with any light sheet modality.

In wavelet transforms, just like the Fourier transform, a signal is described by a set of basis functions and corresponding coefficients. For the Fourier transform, the basis functions consist of sinusoidals with varying frequency, and the coefficients are the amplitude and phase of those functions. The basis functions of a wavelet transform have a set of wavelets $\Psi_{n},n=1,\ldots ,N$ , that function as high-pass filters. One commonly used family of wavelets are the Daubechies (DB) wavelets which exist in various complexity (i.e. increasing vanishing moments, denoted DB2, DB4,…, DBN).

Unlike sinusoids, wavelets are localized in both time (or space) and frequency, which allows them to capture discontinuities and sharp changes more effectively. Thus, the wavelet transform is integrated both in time (or space) and in scale, the latter of which captures different levels of detail. To accomplish this, the basis functions of a wavelet transform also include scaling functions $\Phi_{n}$ , that function as low-pass filters. For discrete transforms, the application of the scaling function translates to distinct levels l=1,…,L , in which the wavelets are applied, such that the signal f(x) at level *L* is approximated by

(3) $$f(x)=\sum_{n}c_{L,n}^{S}\times \Phi_{L,n}(x)+\sum_{l=1}^{L}\sum_{n}c_{l,n}^{H}\times \Psi_{l,n}(x)\;\text{.}$$

Here, $c_{L,n}^{S}$ are the scaling coefficients for level L and $c_{l,n}^{H}$ the wavelet coefficients for each scale. For level L+1 , the scaling coefficients are further decomposed in the same way, halving the amount of coefficients at each level. The scaled wavelets can be derived from the mother wavelet using

(4) $$\Psi_{l,n}(x)=2^{-l/2}\times \Psi_{0}(\frac{x-2^{l}\times n}{2^{l}})\;\text{.}$$

For a 2D signal, such as an image, the wavelets are applied in each dimension separately, as well as diagonally, resulting in three sets of wavelet coefficients at each level: $c_{l,n}^{vert},c_{l,n}^{hor}$ and $c_{l,n}^{diag}$ . By using wavelet decomposition for filtering, vertical stripes of a certain width can be specifically targeted through the damping of vertical wavelet coefficients at the appropriate level. In wavelet-Fourier filtering, this filtering is applied in the Fourier domain of those coefficients, which further isolates the targeted artifacts.

**Fig. 5.**
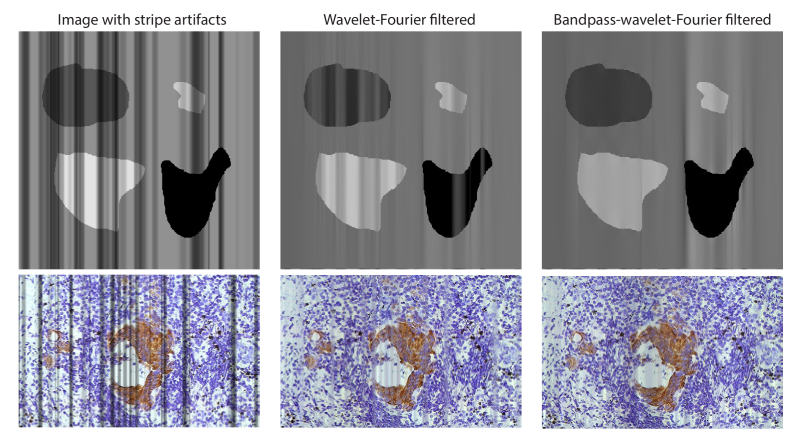
Example images with simulated shadowing stripes in the vertical direction. Using wavelet-Fourier filtering efficiently suppresses lines in the homogeneous background, but introduces lines in the regions of low and high intensity. By adding bandpasses we adapt the filtering to be effective in all intensity regions.

Wavelet-Fourier filtering is effective in images with constant mean intensity, but it can introduce or emphasize hidden line structures in regions of low and high intensity. To mitigate this issue, we modified the filtering scheme with additional additional bandpass steps, creating bandpass-wavelet-Fourier filtering. To demonstrate this, we generated shadow stripes in two sample images (demo images in MATLAB) by attenuating the intensity through super-Gaussian stripes. Figure 5 displays the images processed with wavelet-Fourier filtering and bandpass-wavelet-Fourier filtering.

An image frequency band is created by normalizing the image intensities through division with the local mean intensity before wavelet decomposition. The mean intensity image is created using a small Gaussian kernel that effectively low-pass filters the image. This approach differs from the inherent low-pass filtering within the wavelet decomposition, as calculations are performed on the ratio of the original image to the low-pass filtered image, rather than on the components themselves. Additionally, a different two-dimensional kernel is used for this process. After filtering, the destriped image is recombined with the mean intensity image to restore the original intensity variations.

The approach effectively normalizes the relative strength of the shadowing to the signal strength within a region, assuming the region is larger than the Gaussian kernel. Concurrently, the filter minimizes the suppression of structures larger than the kernel. If it is challenging to find a kernel size that accommodates both the smallest intensity regions and the largest line structures, further image deconstruction can be performed.

**Fig. 6.**
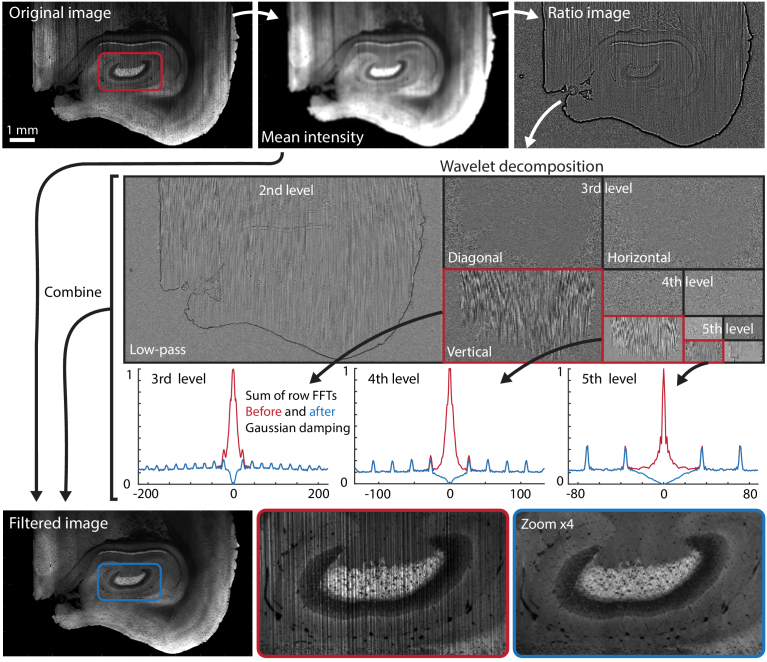
Bandpass-wavelet-Fourier filtering. The original image is separated into frequency band images (here, high and low) before wavelet decomposition of each band image. Selected vertical wavelet components are then subjected to Gaussian damping of low frequencies in the Fourier domain. The filtered image shows efficient shadowing suppression despite strong variations of signal intensity along the lines.

In this process, a larger Gaussian kernel is applied to the initial mean intensity image, creating a bandpass of Gaussian kernels. The wavelet-Fourier algorithm is then applied to the bandpass image. After filtering, the resulting image is combined with the other destriped band images and the mean intensity image. This bandpass-wavelet-Fourier filtering scheme is illustrated in Fig. 6.

For each bandpass, the wavelet decomposition is done using the built-in "dwt2" method, which computes the single-level wavelet decomposition of the image using a specified wavelet and MATLAB-default high- and low-pass filters. The function returns the high-pass, low-pass, vertical, and horizontal wavelet coefficient matrices. Note that high- and low-pass here refer to the wavelet filter. Higher-level decompositions are performed by iterative decomposition of the low-pass component. The four matrices for each level are saved for re-combination after Fourier filtering.

In the next stage, the actual filtering is performed. A high-pass Gaussian damping filter (Eq. (5)) is applied in the Fourier domain of the vertical wavelet components. The filter may be applied to any subset of decomposition levels and the width of the filter, dictated by the *σ* parameter, may be optimized for each frequency band. The filter must be wide enough to properly suppress the spread of frequencies in one decomposition level, including shadowing that is not perfectly vertical.

(5) $$g(x,y)=1-e^{-\frac{y^{2}}{2\sigma^{2}}}$$

The filtered bandpass image is assembled by iterative inverse wavelet decomposition, recreating lower-order wavelet components from the filtered vertical components together with the original high-pass and horizontal components. Next, the full filtered image is reconstructed by multiplying the destriped frequency bands and the last mean intensity image. Finally, by segmenting the tissue using a binary mask, the tissue from the filtered image is merged with the unfiltered background to ensure that no line structures are introduced in the noise outside of the tissue.

### 3.2. Evaluation and optimization

The efficiency of any filtering technique is dependent on the choice of parameters. In order to preserve image information, wavelet-Fourier filtering specifically targets line structures by limiting Gaussian damping to a subset of the information. Fundamentally, however, there is still a trade-off between resolution loss and line suppression. This trade-off is largely controlled by the width of the Gaussian filter and on which wavelet decomposition levels it is applied.

In addition, in our algorithm, the wavelet-Fourier filtering is separated into frequency bands in order to avoid creating artifacts in varied intensity regions. Here, a high frequency band enables the preserved integrity of small areas of low intensity, such as non-fluorescent cell nuclei or small cavities from vascular formations. However, in this band only high frequency line structures can be suppressed. Larger line structures require a lower frequency band in order to suppress. If there are several types of line artifacts of various sizes present in the data, each type of line artifact should match one spectral band. Although there should be little additional information loss from using more bands, increased granularity comes with a trade-off with computation time. For a single high-pass filter, where only high frequencies are filtered, the added time is negligible. For each additional band to filter, the computational time increases linearly. Using our hardware (see section 2.5), the algorithm processed data at 51 GB / hour using a single bandpass, 35 GB / hour using two, and 26 GB / hour with three bandpasses.

**Fig. 7.**
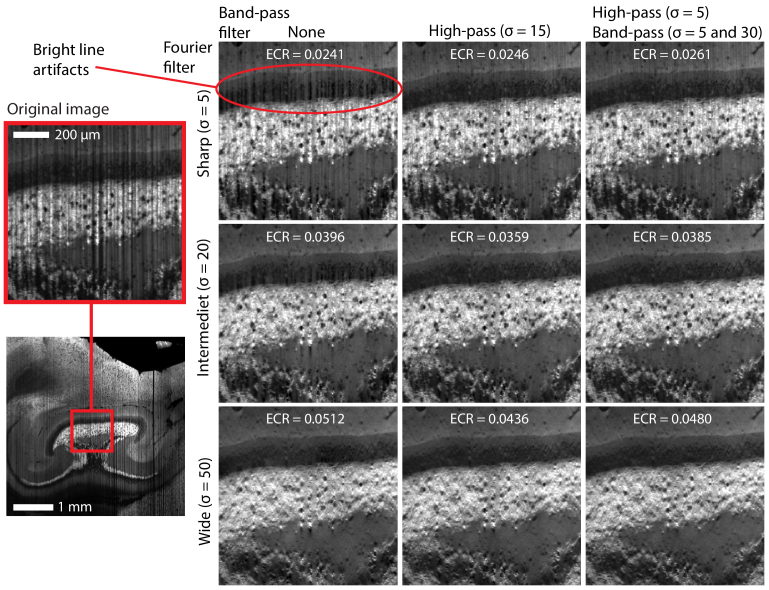
Comparison of filter settings for wavelet-Fourier filtering with bandpass of an image with regions of varying intensity. Bright line artifacts appear in the dark regions. These can be suppressed either by a wider Fourier filter at the cost of reduced image sharpness, or through a high pass filter step. Multiple bandpasses provide a more adaptable filter.

To find suitable settings for the best filtering performance, images were primarily inspected manually. However, a way to quantify the total distortion of the image and avoid excessive filtering is to calculate the total energy change ratio through

(6) $$ECR=\frac{\sum_{pixels}{\left(I_{o}-I_{f}\right)}^{2}}{\sum_{pixels}I_{o}^{2}}\;\text{,}$$

where $I_{o}$ and $I_{f}$ are the original and filtered image, respectively. In Fig. 7, an image of part of the cortex and hippocampus with strong intensity differences is shown for various combinations of Gaussian filter sizes for the Fourier filter and the frequency bands.

It is clear that the filtering is highly effective, as visible artifacts are significantly reduced compared to the original image in all cases. With a sharp Fourier filter, some residual lines persist regardless of bandpass filter. Clearly, here, the frequency spread of the artifacts are wider than the sharp filter. With no bandpass filter, strong filter artifacts have appeared in the low intensity region. When introducing a high- or bandpass, these bright lines are efficiently removed.

For the high pass, a rather large filter is needed to capture the broadest lines. Thus, bright line artifacts appear in the varied region in the bottom of the image. By using a higher cut-off for the high pass filter with an additional bandpass these bright lines can be suppressed. Introducing a third bandpass does not give any obvious improvements in this case, but may be useful at a different resolution.

By increasing the width of the Fourier filter to the intermediate size, the full spread of the original shadowing can be suppressed. This also smooths out the new line artifacts in the low-intensity regions. However, this increases the distortion of the image, which is evident from the higher ECR values. With a wide Fourier filter there are no longer any bright line artifacts even without a band-pass filter, though this comes at the cost of visibly reduced resolution.

In the comparison in Fig. 7, for the sake of rigidity, the width of the Fourier filter, the targeted wavelet decomposition levels, and the wavelet transform used are constant for each case. For this comparison, we used the Daubechies 45 wavelet. In practice however, all these filter settings can be optimized for each decomposition level and frequency band combination, potentially yielding even better results. Although exact values will depend on the specific image data, Table 1 summarizes the heuristics to determine a good starting point for each parameter.

**Table 1.** Summary of modifiable parameters for the shadowing artifacts suppression algorithm and how to approximate good values for each. Variable names correspond to the names in Code 1, Ref. [39].

**Variable name**	**Definition**	**Approximation heuristics**

**gauss_sizes**	2D Gaussian filter sizes for bandpasses. Tuple with N values.	Suggested number of bandpasses (N) is the types of stripe artifacts + 1
gauss_sizes{1}	1:st high pass filter	Pixel size of smallest dark region to preserve
gauss_sizes{2}	2:nd high pass filter	Pixel width of smallest stripe artifact type
gauss_sizes{N}	N:th high pass filter	Pixel width of (N-1):th smallest stripe artifact type
**dec_levels**	Wavelet decomposition levels to filter. Tuple with N integer arrays or scalars.	
dec_levels{1}	Array of levels for 1:st bandpass	2 to log_2_(gauss_sizes{1})(level 1 is often not necessary)
dec_levels{2}	Array of levels for 2:nd bandpass	1:st filter size to log_2_(gauss_sizes{2})
dec_levels{N}	Array of levels for N:th bandpass	(N-1):th filter size to log_2_(gauss_sizes{N})
**wavelet**	Name of wavelet to use	High order DB wavelet suitable, such as ‘db45’
**sigma**	Width of Gaussian filter in Fourier domain	Deduced from width of central peak in fft of wavelet decomposition. Approximate automatically through syntax: “*bandpass_wavelet_fourier(my_image, 15, 2:3, ‘db45’, **-1**)”*

The higher-order Fourier peaks visible in Fig. 6 are likely to be residuals from the sinusoidal period of the structured light sheet. After the Gaussian damping of low frequencies at multiple wavelet decomposition levels, residuals from these are no longer visible. Even so, attempts were made with a multi-Gaussian filter that also targeted the higher-order Fourier peaks. However, this did not yield any visible improvements but did increase the total energy change in the filtered image, and thus this was not pursued further.

## 4. Light extinction compensation

### 4.1. Theoretical background

Significant signal attenuation in the original data volumes is caused by multiple light scattering and absorption of the excitation light while moving through the tissue. The effect is particularly noticeable in the SILMAS data, as a result of the effective elimination of scattered light. In fact, this makes SILMAS data well suited for compensating for attenuation in a nearly quantitative manner, ensuring consistent intensities across the entire sample.

In cases where the optical properties are relatively uniform within the tissue, the attenuation along the propagation direction will adhere to an exponential decline, as described by the Beer-Lambert law. Because unsaturated fluorescence is proportional to excitation intensity, the fluorescence emitted along this direction will display the same decay. The emitted fluorescence will then experience attenuation along its new propagation direction following another exponential decay. Thus, the fluorescence intensity from a small volume $dx^{3}$ at tissue depth (x,y,z) , observed perpendicular from the light sheet, in a sample with homogeneous fluorescence and extinction can be described through

(7) $$I(x,y,z)=I_{0}(y,z)e^{-\mu (\lambda_{1})x}\times \Phi_{f}\mu_{a}(\lambda )dx\times e^{-\mu (\lambda_{2})z}\;\text{, where}$$

(8) $$\mu (\lambda )=\mu_{a}(\lambda )+\mu_{s}(\lambda )\;\text{.}$$

Here, $I_{0}(y,z)$ is the incident illumination intensity, *μ*, $\mu_{a}$ , and $\mu_{s}$ are the extinction, absorption, and scattering coefficients at the excitation and fluorescence wavelengths $\lambda_{1}$ and $\lambda_{2}$ . $\Phi_{f}$ is the fluorescence quantum efficiency.

From a pathology perspective, the fluorescence signal of interest is the sparsely distributed high-intensity aggregates of SynGFP. Here, in contrast, we focus on the surrounding tissue that is characterized by relatively homogeneous fluorescence of lower intensity from endogenous SynGFP. This signal can be described using Eq. (7), and by quantifying the extinction from this signal. Our extinction compensation algorithm in Code 1, Ref. [39] is implemented through the following steps:

• Segment the tissue within the image volume using a binary mask.
• Map voxel coordinates (x′,y′,z′) to the corresponding depth of penetration (x,z) .
• Compensate data for illumination variations $I_{0}(y,z)$ .
• Extract the relationship between the penetration depth for the excitation(*x*), fluorescence (*z*), and intensity I(x,y,z) .
• Fit the normalized extracted relationship to the 2D exponential decay (Eq. (7)).
• Compensate all voxel values based on their position within the tissue.

Other than very effective elimination of light scattering, this scheme relies on volumetric data recorded with single sided illumination. The data also need to include the sample border in both the X- and Z-axis.

### 4.2. Tissue segmentation

**Fig. 8.**
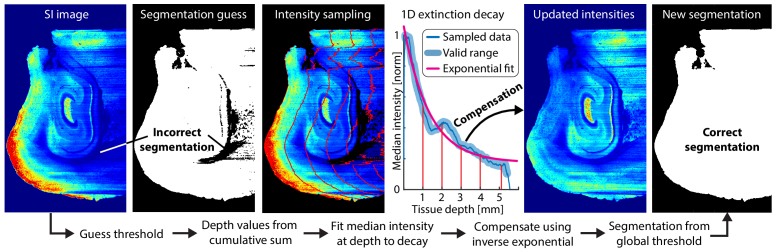
Pre-compensation for improved tissue segmentation of tissue with significant attenuation. An initial intensity threshold is used to create a depth map of the tissue (X-axis). The median intensity value is extracted for each depth from which an exponential fit is calculated. The inverse function is then used to compensate for the attenuation, and an improved intensity threshold is used to create the mask.

Segmenting the tissue is a prerequisite for compensating for extinction. However, finding a global threshold is challenging in cases of high attenuation, as the signal may be weak and noisy at higher depths. To solve this, we pre-compensate for extinction in each Z-plane when creating the segmentation mask in Code 2, Ref. [40]. The procedure for this follows the same logic as for the volumetric data in extracting the exponential decay, and is illustrated in Fig. 8.

In the pre-compensating scheme, the Z-plane undergoes an initial noise suppression by applying a Gaussian filter with a radius of 5. Following this, an initial mask is generated by applying a global threshold to the filtered image. The cumulative sum of the mask along the light propagation direction (X-axis) is calculated to define a propagation depth map, from which the median pixel value at each depth is derived.

The resulting array is trimmed to remove values below the original threshold, normalized, and fitted to an exponential decay function. This decay function is applied to the depth map to create an extinction map, by which the filtered plane is divided. To refine the adjustments, the depth map is updated by reapplying the threshold, subjecting it again to the exponential decay, and generating a new extinction map. This updated extinction map is then used to produce the pre-compensated plane.

From the pre-compensated plane, a global threshold can be found by analysing the image histogram. Here, the first zero corresponds to the threshold between the background signal and the fluorescence of the endogenous tissue. A series of morphological operations are then performed on the segmentation to close small gaps and even the edges.

In our image processing pipeline, this segmentation procedure is performed on each image in 2D. However, when considering a stack of images that are segmented in order, the initial guess can be replaced by the finished segmentation from the previous image. Additionally, the adjacent image can be used as a probability weight for pixels values close to the threshold. Thus, the likelihood of including a pixel will be increased if its Z-axis neighbour is segmented as tissue. With our hardware (see section 2.5), the segmenting algorithm processed data at 63 GB / hour.

### 4.3. Calibration and application

From the 3D stack of 2D segmentation masks, we can map the voxel coordinates to the depth of penetration of the tissue on the x and z axes. In the 2D extinction adjustment, the cumulative sum of the mask could define an approximate depth. However, when accounting for the numerical aperture of the incident light sheet and the imaging optics, the true penetration depth is higher than the axial distance. The NA of the objective corresponds to a collecting cone with half-angle of about 10 degrees, which was approximated by introducing a cumulative circular blur (r = 1) in the Z-axis of the mask. The NA of the incident light is lower and was neglected, again defining the X-axis depth by the cumulative sum of the mask.

Before mapping voxel values to the depth positions they need to be compensated for illumination variations. The Y-axis profile of the incident light sheet was measured beforehand by extracting the signal from a homogeneous mixture of mounting media and fluorescein. Additionally, our measurement was optically pre-compensated in the Z-axis by using a linear incremental increase of illumination power to increase signal strength. To calculate real extinction in the Z-axis this pre-compensation needed to be accurately accounted for.

**Fig. 9.**
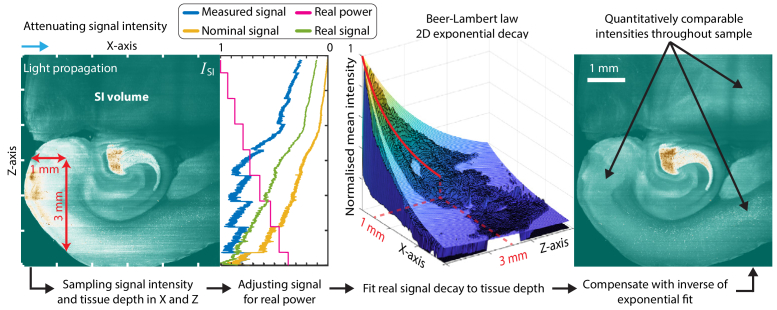
Light extinction compensation in 3D visualized by a X-Z maximum intensity projection over 1 mm in Y-axis. Illumination power changes in the Z-axis are compensated for using the mean signal intensity of each XY plane. A 2D exponential is fitted to mean intensities for each X- and in Z-penetration depth and used to compensate attenuation.

The OBIS lasers’ drive voltage are calibrated to chosen nominal output power. The real output power was not measured during acquisition, but, rather than naively using the nominal output power, a data approach was used. Using the binary mask, the mean signal from the tissue was calculated for each Z-plane. By comparing a moving mean of 20 planes forwards and backwards, the effect of the illumination power shifts could be isolated. We found a mismatch between real and nominal shifts, primarily at low laser powers. This caused the pre-compensation to be less effective than expected, linearly increasing illumination power with about 160% instead of the intended 400% over the range of the scan.

With the real signal of each voxel, I(x′,y′,z′) , and the coordinate mapping in place, we can finally define the extinction relationship. The normalized mean values $\frac{1}{N}\sum_{y}I(X,y,Z)$ for X and Z ranging the full depth of the volume are shown in Fig. 9. The data are fitted to a 2D exponential decay by which the voxel values are compensated. The result is shown to the right in the figure. The compensation algorithm (excluding segmentation) processed the data at 47 GB / hour using our hardware (see section 2.5).

## 5. Results and discussion

**Fig. 10.**
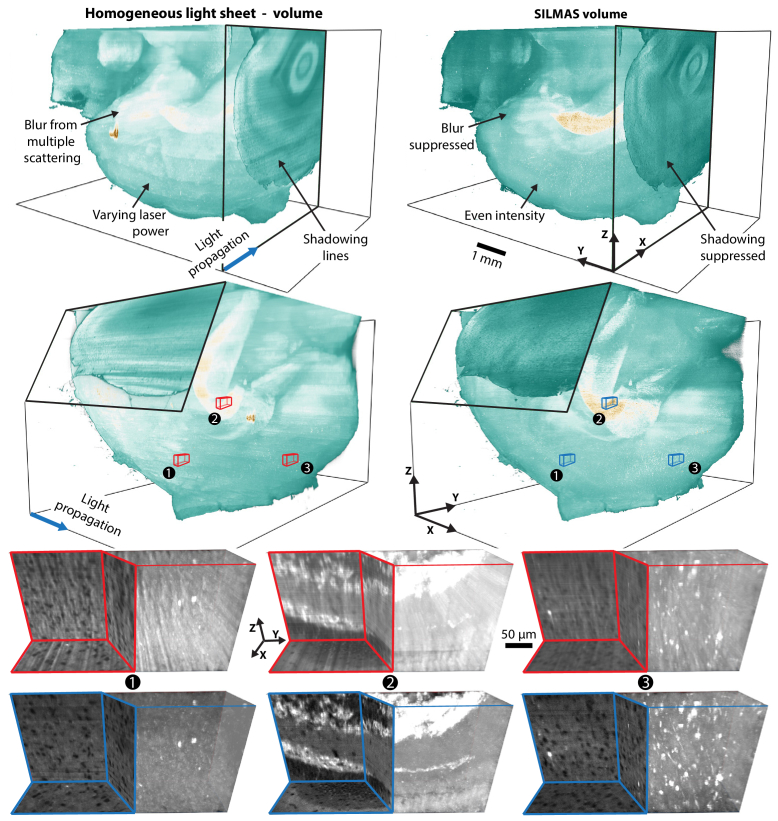
TOP: Imaging improvements from the full SILMAS procedure after utilising structured information, shadowing suppression and extinction compensation. Shown from two angles. BOTTOM: Zoom-ins of regions at various light penetration depth, shown as both planar sections and volumetric data.

This multi-step filtering pipeline is custom fitted and intended to use with volumetric SILMAS data. Thus, to evaluate performance, it is useful to visualize the improvements from the full pipeline in 3D. Figure 10 shows one full volumetric image tile stack, before and after all data improvements from the imaging pipeline: scattered light suppression, extinction compensation, and shadowing suppression.

Before improvements, the data suffer from unevenly bright regions, bright blur from scattered light, and line structures in the X-axis. In contrast, the SILMAS volume shows even intensity, improved contrast, and the shadowing lines are significantly suppressed. The hippocampus, which is visible as a bright region in the center of the volume, is distinct, uniform, and anatomically representative after improvements, while it was distorted before.

In the lower half of Fig. 10 are zoom-ins of three regions at varying depth in the larger volumes. In region 1, which is at the lowest tissue depth, the contrast in both volumes is relatively high. However, strong line structures limit the visibility in the homogeneous volume. In regions 1 and 2, the contrast is severely reduced in the homogeneous volume, and a loss of resolution akin to optical smoothing is seen in the Z-axis. Meanwhile, the SILMAS volume maintains the high contrast and integrity of the optical sectioning. Thus, the filtered volume has nearly seamless integration and uniform resolution in 3D.

Although it is clear that the imaging and processing described here provide many of the desired improvements, some choices have been made to simplify the post-processing pipeline considering the large amount of data. The complexity level of the filtering strategies used allowed us to perform the post-processing on a moderately powerful desktop PC. With our hardware (see section 2.5) and standard settings, the processing speed for the full scheme is 15 GB / hour, but depends on the number of bandpasses used. Additional filtering for noise suppression, or sharpening of specific features can easily be added to the scheme.

For the shadowing suppression, it is possible to expand the filtering into 3D. However, this approach is unlikely to yield significant advantages for several reasons. First, shadowing is not a strong effect in the Z-direction, as fluorescence is isotropic and the NA of the collection optics is typically higher. Second, while sampling in 3D, light sheet imaging is fundamentally a 2D imaging technique. As such, noise and artifacts are only continuous within the plane. Third, the benefit of wavelet filtering is to isolate the artifacts, which are in 1D, and avoid deteriorating the data in other dimensions. Finally, to overcome the computational strain of 3D filtering it would likely have to be done in smaller subsets of data, subverting the goal of continuous connectivity.

For the light extinction compensation, the underlying assumption from using a single segmentation for the entire mouse brain is that the extinction coefficients are invariant to brain region. For the absorption coefficient, it is evident from the intensity difference between regions that this is not the case, with distinct difference between, e.g., the hippocampus and cortex. However, a large part of the extinction is due to multiple scattering, as indicated by the large difference in attenuation between the homogeneous and SI volumes (see Fig. 3). In uncleared brain tissue the assumption of constant scattering between regions would be far fetched; however, after clearing the tissue is more homogeneous. In total, the emergence of an exponential decay with low residuals for the whole volume validates the assumption of mostly invariant extinction.

At high penetration depths, the SNR quickly drops to low levels. The signal here is weak due to accumulated light extinction and compensating for this produces more noisy data. For a pipeline that also includes noise suppression, it is prudent to do this before applying the compensation. Similarly, to not highlight noise within shadowing lines, shadowing suppression should also be applied before extinction compensation. Note that this concerns applying the compensation, not calculating the extinction calibration, which has integrated averaging.

In more deeply located anatomical regions, such as the midbrain, optical properties may differ from the outer regions due to difficulty of clearing evenly through thick tissue. With the bulk of the sample being the cerebral cortex, whose optical properties appear relatively homogeneous, the global estimation of the optical properties will be most influenced by the values of this region. Consequently, this near-quantitative compensation of intensities will also be most accurate in the cortex. To improve the attenuation compensation, the data could be separated into distinct anatomical regions before extinction estimation. This can either be made manually or automatically via mapping the volumes to a mouse brain atlas. Then, the same extinction estimation approach can be applied on a region-by-region basis. Clearly, such an approach comes at the cost of higher complexity.

## 6. Conclusion

There are clear benefits of SILMAS imaging in providing higher-contrast data and improved optical segmentation on a large FOV. However, it produces data sets with strong intensity variations which limits the methods usefulness for visualization and data analysis. The proposed filtering adds to the benefits of the imaging by providing comparable intensities throughout the entire sample. This is achieved by suppressing shadow artifacts and line structures and by compensating for light attenuation.

Each step of the imaging pipeline has a distinct purpose: light scattering suppression, extinction compensation, and shadowing suppression. The post-imaging steps are valid for any LSFM modality with optical light scattering suppression. However, when the composite steps are brought together in SILMAS, there is a synergy of the full method. The extinction compensation is only possible thanks to optical light scattering suppression, computational shadowing suppression is more effective with high-NA axial sweeping, and wavelet-Fourier filtering targets both shadowing and structured illumination residuals.

Wavelet-Fourier filtering is an effective method for removing line structures in LSFM data. However, in a FOV covering regions of diverse intensity with high contrast, significant artifacts of the filter are evident in areas of low intensity. By implementing a high- or bandpass filter, these pronounced lines can be effectively eliminated without degradation of the image. Using more than two bands provides limited improvements at this resolution while adding significant processing time. Bandpass-wavelet-Fourier filtering is effective for SILMAS and can also be used for other types of light sheet images.

The attenuation compensation produces an image volume which has comparable intensities at different tissue depths, particularly within the same brain region. The tissue-wide approach makes intensity compensation most accurate in the bulk of the imaged tissue. To improve the extinction calibration, the data could be separated into distinct anatomical regions prior to extinction estimation. To avoid highlighting low SNR in low light regions, shadowing and noise suppression filtering should be applied before the attenuation compensation.

Ultimately, the objective of applying volumetric imaging to neurodegenerative research in murine models is to quantitatively analyse the spatial distribution, size, and characteristics of pathological features with greater accuracy within the 3D environment. To this end, refined SILMAS data featuring uniform intensities across the sample, along with strong contrast and precise optical segmentation, will provide a significantly improved starting point for subsequent analysis.

## Data Availability

A subset of the data underlying the results presented in this paper is available in Dataset 1 [38]. The full data are not publicly available at this time but may be obtained from the authors upon reasonable request.
